# Supplementary figures and images for: Efficient Genome Editing in Bacillus licheniformis Mediated by a Conditional CRISPR/Cas9 System
Source: Microorganisms. 2020 May 17;8(5):754. doi: 10.3390/microorganisms8050754 (PMC7285353; doi:10.3390/microorganisms8050754)

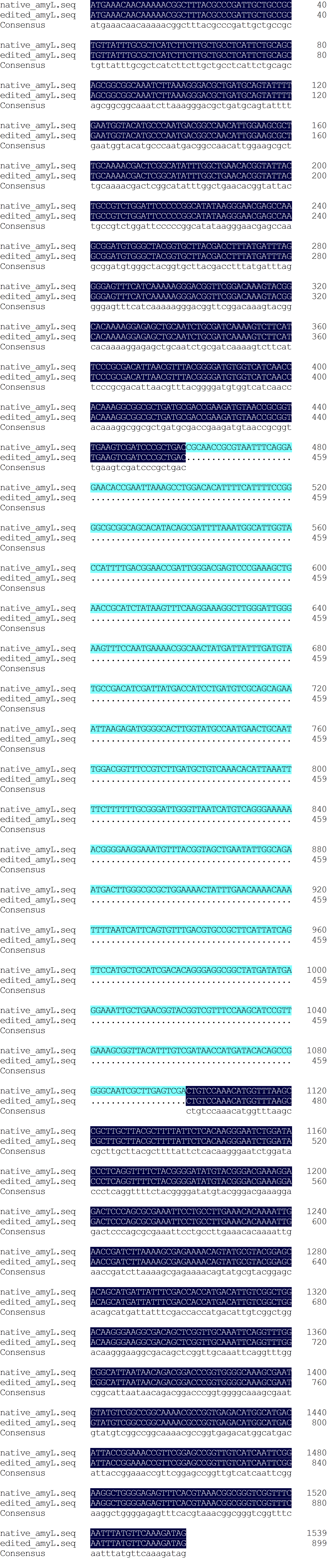

Supplement: Supplementary file 1 [file microorganisms-08-00754-s001.zip › Supplementary Figure 1.jpg]
